# The influence of leg-to-body ratio, arm-to-body ratio and intra-limb ratio on male human attractiveness

**DOI:** 10.1098/rsos.171790

**Published:** 2018-05-16

**Authors:** Thomas M. M. Versluys, Robert A. Foley, William J. Skylark

**Affiliations:** 1 University of Cambridge, Cambridge, UK; 2 Leverhulme Centre for Human Evolutionary Studies, University of Cambridge, Cambridge, UK; 3 Department of Psychology, University of Cambridge, Cambridge, UK

**Keywords:** attractiveness, morphology, limb proportions

## Abstract

Human mate choice is influenced by limb proportions. Previous work has focused on leg-to-body ratio (LBR) as a determinant of male attractiveness and found a preference for limbs that are close to, or slightly above, the average. We investigated the influence of two other key aspects of limb morphology: arm-to-body ratio (ABR) and intra-limb ratio (IR). In three studies of heterosexual women from the USA, we tested the attractiveness of male physiques that varied in LBR, ABR and IR, using figures that ranged from −3 to +3 standard deviations from the population mean. We replicated previous work by finding that the optimally attractive LBR is approximately 0.5 standard deviations above the baseline. We also found a weak effect of IR, with evidence of a weak preference for the baseline proportions. In contrast, there was no effect of ABR on attractiveness, and no interactions between the effects of LBR, ABR and IR. Our results indicate that ABR is not an important determinant of human mate choice for this population, and that IR may exert some influence but that this is much smaller than the effects of LBR. We discuss possible reasons for these results, including the limited variability in upper limb proportions and the potentially weak fitness-signal provided by this aspect of morphology.

## 1. The influence of leg-to-body ratio, arm-to-body ratio and intra-limb ratio on male human attractiveness

Attractiveness has a substantial impact on human well-being, with more attractive people being perceived as more sociable [1], intelligent [2] and healthy [3] than their less attractive counterparts. This set of positive attributions, known as ‘the halo effect’ [4], arguably has significant implications for life outcomes, because attractiveness is positively correlated with job prospects [5,6], wages [7], judicial decisions [8], election results [9,10], marriage stability [7,11] and biological fecundity [12]; attractiveness may also be *disadvantageous* in some contexts (e.g. [13]).

One key determinant of attractiveness is body morphology, and explorations of the link between morphology and attractiveness go back at least as far as Leonardo da Vinci's Vitruvian Man. From the perspective of evolutionary biology, attractiveness judgements reflect the biological fitness of a prospective mate (i.e. their ability to survive and reproduce in their environment), because a high-fitness mate is likely to be better able to provide resources, care, and protection, to be less likely to transmit harmful pathogens, and to pass on ‘good genes’ to the offspring [14]. Because some aspects of fitness correlate with morphological traits, numerous studies have investigated how a variety of prominent anatomical features influence attractiveness, including face shape [15], body fat percentage [3] and height [16]. In the present paper, we investigate a relatively unexplored but potentially crucial aspect of morphology: limb proportions.

Previous studies of the link between limb proportions and attractiveness have focused on leg-to-body ratio (LBR), defined as the ratio of total leg length to total height. Two early papers reported a preference for low LBRs [17,18], but these experiments used line-drawn figures with anatomical distortions in the hands, feet and crotch introduced through crude image-manipulation techniques. Subsequent work has typically used silhouettes based on photographs and has found a preference for average or above-average LBRs [19,20] (see also [21–23]). Some researchers have suggested that silhouettes lack ecological validity and may not accurately represent human body morphology [24,25], and more recent research has progressed to using naturalistic 3D-rendered CGI figures [26,27]. In a study of Japanese participants, Kiire [27] examined a wide range of LBRs using realistic rendered figures and, using a curve-fitting approach, found that the optimal attractiveness was very close to the population mean; in a similar study, Versluys & Skylark [25] found that, for heterosexual American women, the optimally attractive LBR for rendered male figures was approximately half a standard deviation above the population average. (These authors also found that the effect of LBR was different for 3D-rendered images and silhouettes, confirming that studies of silhouette figures should be treated with some caution.)

These findings have been explained in terms of the fitness associated with different LBRs. ‘Averageness’, defined as the degree to which a trait approximates the mean of that trait in the population [15], is believed to reflect genetic diversity which, in parts of the genome coding for immunocompetence, reduces the chance of being targeted by locally specialized pathogens [28,29]. In addition, LBRs that are slightly above the mean are associated with high socioeconomic status, good nutrition and developmental stability [30], as well as biomechanical efficiency during locomotion [31,32]. High LBRs are also linked with larger overall stature (because leg length is the main contributor towards height increases beyond the average), which suggests that LBR can serve as a proxy for overall size [14]. In contrast, significant deviations from the average, in either direction, are associated with poor health outcomes: short legs are linked to insulin resistance syndrome and type 2 diabetes [33,34], coronary heart disease [33,35,36], high blood pressure [37], and dementia [38,39], while extremely high LBRs often indicate deleterious genetic conditions, such as Marfan syndrome [40]. The observed preference for LBRs that are at or slightly above the population mean therefore accords with the idea that these LBRs signal the fitness of a prospective mate.

The present research builds on these studies by investigating two aspects of limb morphology that have not previously been studied: arm-to-body ratio (ABR) and intra-limb ratio (IR).

ABR is defined as the ratio of total arm length to total height. Although research is relatively sparse, short arms have been associated with negative mental health outcomes, especially Alzheimer's disease/dementia [41–43]. They are also linked to developmental stressors, including cold exposure, hypoxia and malnutrition, as well as poor socioeconomic status [30,44,45], all of which are typically maladaptive. Conversely, there is some evidence that longer arms improve throwing ability [46], which is argued to have aided ancestral activities including hunting and interpersonal conflict [47], although the importance of ABR relative to other anthropometric traits is unclear [48].

IR is defined as the ratio of the distal (i.e. lower) limb segment to the proximal (upper) limb segment. Differences in the distal limb segment account for most of the variation in total limb length [14], so IR might be used as a proxy for overall limb size (i.e. for LBR and ABR) which, as described above, is linked to health. In addition, short distal limbs are associated with developmental stress and socioeconomic deprivation [30,44,45,49]. There are competing explanations for these effects, including the idea that shorter distal limbs represent an adaptation to the cold by reducing heat loss [45], the idea that distal limbs are more susceptible to reduced blood flow [50], and the ‘thrifty phenotype’ hypothesis under which certain tissues are sacrificed to ensure that more vital organs are preserved [30,51]. Whatever the cause, distal limbs are thought to be especially susceptible to environmental challenge [44], and thus IR is likely to signal fitness.

Taken together, these results, coupled with the general principle that averageness signals immunocompetence, suggest that, like for LBR, the optimally attractive ABR and IR will be at or slightly above the population means. However, to date no attempt has been made to explore how these aspects of limb morphology affect attractiveness.

The present research consists of three related studies that address this gap in the literature by examining the effects of LBR, ABR and IR on the attractiveness of male figures to heterosexual women. In Study 1, we manipulate all three limb variables in a factorial design. In Study 2, we expand the set of tested values for LBR, ABR and IR and examine each of the three variables individually. In Study 3, we probe the effects of IR in more detail by examining the effect separately for arms and legs. All three studies implement recent methodological improvements to the study of limb proportions: the ecological validity of the stimuli is improved by using a validated anthropometric database and selecting limb length increments that correspond to a certain number of standard deviations above/below the population mean; stimulus realism is enhanced through the use of sophisticated design software, although the figures are rendered in greyscale to minimize any potential interactions between limb preferences and ethnic background; and a curve-fitting approach is used to estimate optimum limb proportions and the sensitivity to departures from that optimum.

## 2. Study 1

Study 1 examined the effects of LBR, ABR and IR, and whether these different limb proportions interact to shape attractiveness. For each limb ratio, we tested values that were a given number of standard deviations above/below the population mean. This approach has the advantage that the chosen values relate directly to the frequency of particular proportions in the population; it also ensures that the manipulation is comparable across the different ratios. Because this study is the first to investigate the effects of ABR and IR, and because it uses a factorial design, we chose values that were relatively extreme to maximize the chance of detecting an effect and to keep the number of stimulus combinations manageable, while ensuring that the values were within the population distribution. We therefore presented each participant with 25 figures obtained by factorially combining 5 ABRs (−3, −2, 0, +2 and +3 s.d. from baseline) with 5 LBRs (again, −3, −2, 0, +2 and +3 s.d. from baseline). Each participant was tested with one of three IRs (−3, 0 or + 3 s.d. from baseline); IR was varied between subjects to avoid overburdening participants, and only 3 IR values were tested in order to limit the total required sample size. We varied IR within subjects, and used more IR values, in Studies 2 and 3.

### 2.1. Methods

#### 2.1.1. Participants

Participants in all studies were heterosexual women from the United States who were recruited via Amazon's Mechanical Turk, an online platform that approximates the US population more closely than many convenience samples [52] and that produces data of comparable quality to laboratory studies [53]. Participants were tested online (e.g. [25]), and the inclusion criteria were as follows: female, heterosexual, aged 18 or over, who reported no problems with viewing the experimental stimuli and whose IP address had not occurred earlier in the experiment or experimental series (i.e. who had not previously participated in the present or closely related studies). In Study 1, the sample comprised 341 women aged 19 to 76 (*M* = 39.02, s.d. = 12.78), 79.8% of whom identified as White (i.e. White American or White Other), 8.5% as Black (i.e. Black/African American or Black Other), 6.7% as Asian and 5.0% as any other ethnicity. The sample size of the present experiment gives more than 99% power to detect a modest effect ($\eta_{p}^{2}=0.05$; previous studies of limb proportions have found much larger effects than this) for all three variables (IR, LBR and ABR; here and throughout, power calculation was computed using GPower using default assumptions regarding sphericity and correlation between repeated measures [54]).

#### 2.1.2. Stimuli

The stimuli were realistic CGI human male figures created using state-of-the-art design software (Daz Studio 4.9: https://www.daz3d.com/daz_studio) with the Male Anatomy add-on package. This software provides a default, anatomically accurate model (‘Michael 3’) with skeletal dimensions that can be modified precisely to allow fine control over limb proportions. These proportions were selected using a database of male participants derived from the 1988 US Army Anthropometry Survey (ANSUR) [55], which provides 132 standard anthropometric measurements from approximately 9000 US military personnel. Since there are no definitive rules in anthropometry regarding how limb lengths are defined, appropriate measurements were selected from the database based on design practicality and frequency of use in the literature.

#### 2.1.3. Anatomical measurements

Total leg length was measured as the height to the trochanter landmark on the hip minus ankle height, which was calculated as the distance from the floor to the lateral malleolus landmark on the ankle. Anatomical leg length is defined as the length of the tibia plus femur [14], so our approach is likely to give a closer approximation of anatomical leg length than does the more common measurement of distance from base of foot to the perineum (crotch) seen in the literature (e.g. [20,27]). The LBR was calculated by dividing leg length by total height (measured from the base of the heel to the top of the head). The mean LBR was 0.491 with a standard deviation of 0.015.

Total arm length was calculated as the distance from the acromial process on the shoulder to the radial styloid process on the wrist. This measurement corresponds closely to arm length as defined in terms of its constituent long bones and is more anatomically accurate than the common ‘wingspan’ measurement, which measures the distance between the fingertips of the outstretched arms [56], offering a convenient but relatively crude approximation of arm length that includes both bi-acromial breadth (shoulder width) and the length of the hands. The ABR was calculated by dividing arm length by total height. The mean ABR was 0.349 with a standard deviation of 0.010.

IR was calculated by dividing the lower segment of each limb by the upper segment. The lower leg was measured as popliteal height minus ankle height. Of the measurements in the database, this was the closest to anatomical averages for the lower leg established by measuring disarticulated human bones. The upper leg was calculated as total leg length minus lower leg length. The lower arm length was measured as the distance from the medial epicondyle on the elbow to the styloid process on the wrist, while the upper arm was measured as the distance between the acromial process on the shoulder and the distal end of the humerus, marked by the medial epicondyle. Both measurements are widely used in traditional anthropometry. The mean IR was 0.743 (s.d. = 0.033) for the legs and 0.817 (s.d. = 0.032) for the arms.

#### 2.1.4. Stimulus construction

**Figure 1.**
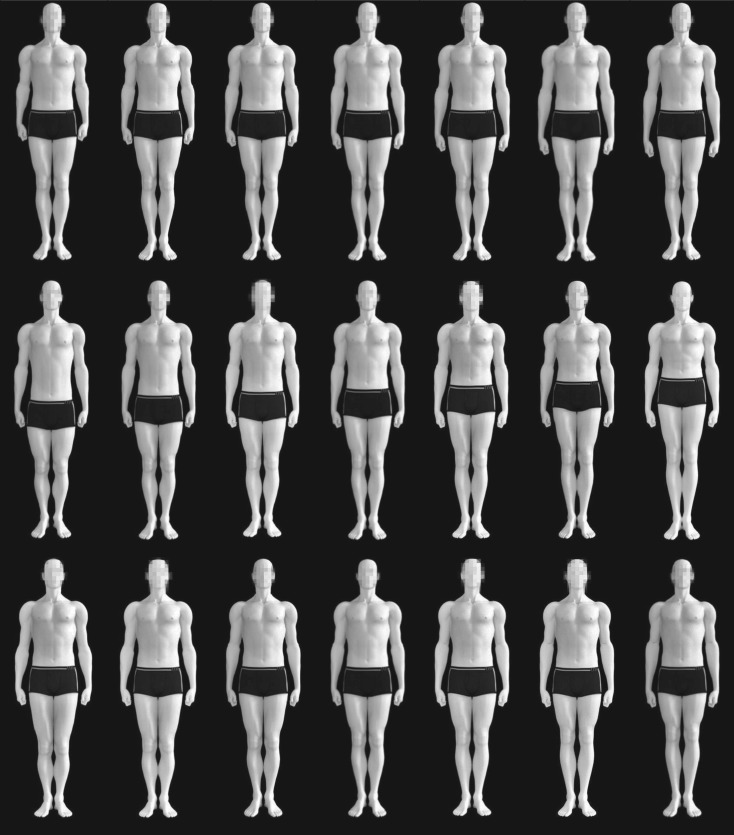
Example stimuli from Studies 1 and 2. From left to right, each row shows figures that are −3, −2, −1, 0, +1, +2 and +3 from the population mean (baseline). The top row shows variation in the ABR; the middle row shows variation in the LBR; the bottom row shows variation in the IR (with changes for both arms and legs).

A baseline figure was created using the proportions of the original model provided in Daz Studio (‘Michael 3’), with nominal height set at the database male average of 175 cm. Limb proportions were set at the database averages of 0.491 for LBR, 0.349 for ABR, 0.743 for leg IR and 0.817 for arm IR. Additional stimuli were created with LBRs and ABRs that were ±2 or 3 s.d. from the mean and IRs that were ±3 s.d. from the mean. These were combined in a full-factorial design to produce a total of 75 unique stimuli. IR was treated as a single variable with concomitant changes in the legs and arms (e.g. a value of IR +1 means that both the leg IR and the arm IR were 1 s.d. above their respective means). The stimulus values are listed in table 1. The faces were pixelated using Lunapic (https://www196.lunapic.com/editor/) [25]; pixilation was applied separately for each figure, resulting in some (non-systematic) variation between stimuli. Images were saved as jpeg files; example stimuli are shown in figure 1.

**Table 1.** Tested values of LBR, ABR and IR.

stimulus	LBR	ABR	IR (legs)	IR (arms)
−3 s.d.	0.447	0.317	0.644	0.721
−2 s.d.	0.462	0.328	0.677	0.753
−1 s.d.	0.477	0.339	0.710	0.785
0	0.491	0.349	0.743	0.817
+1 s.d.	0.506	0.359	0.776	0.851
+2 s.d.	0.521	0.370	0.809	0.882
+3 s.d.	0.535	0.380	0.841	0.914

#### 2.1.5. Design and procedure

The study had a 5 (LBR) × 5 (ABR) × 3 (IR) design. Each participant was assigned randomly to one IR group (−3 s.d., 0 s.d. or +3 s.d. from the population mean), which was a between-subject factor (*N*_−3s.d._ = 112; *N*_0s.d._ = 117; *N*_+3s.d._ = 112); participants judged all combinations of LBR and ABR for their given IR, for a total of 25 figures.

The first page of the task asked the participant's gender; those who responded ‘male’ were directed away from the survey. After providing informed consent, participants were notified that they would be asked to judge the attractiveness of male figures. They were told that some of the figures were similar to one another but that they were all slightly different, that there were no right or wrong answers, and that they should answer honestly. Each figure was presented on a separate webpage and participants rated its attractiveness on a scale from 1 (‘not at all’) to 7 (‘very much so’). Stimulus order was randomized. At the end of the study, participants reported demographic information: ethnic origin (White American; White other; Black/African American; Black other; Hispanic; Asian; Native American; Pacific Islander; Other); sexuality (Straight or heterosexual; Gay or lesbian; Bisexual; Other; Prefer not to say); and age (indicated with a slider ranging from 0 to 100).

### 2.2. Results and discussion

**Figure 2.**
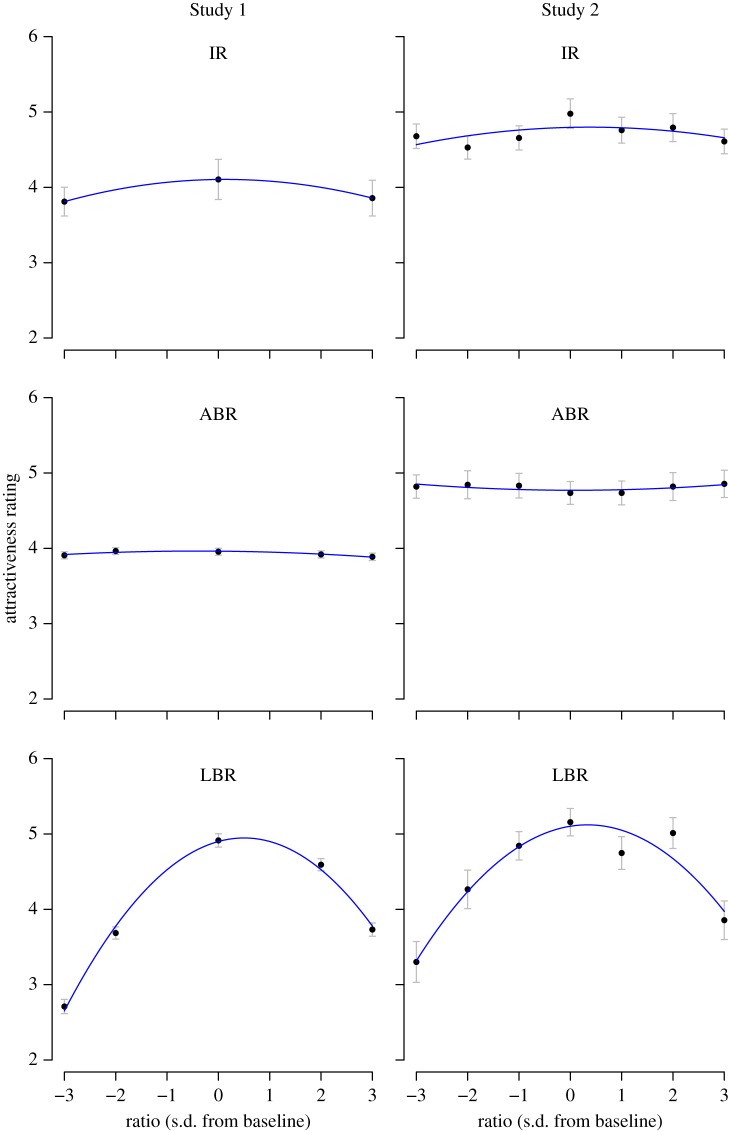
Results of Studies 1 and 2. The plotted points show the mean attractiveness rating for each condition; error bars show 95% confidence intervals, computed for a within-subject design where appropriate [57]. The blue lines show the best-fitting quadratic curves.

The mean attractiveness ratings are plotted in the left column of figure 2. There was little effect of variation in the ABR, *F*_41_ _352_ = 1.89, *p* = 0.110, $\eta_{p}^{2}=0.006$. Judgements did depend on the IR, although the effect was not especially strong, *F*_2, 338_ = 3.35, *p *= 0.036, $\eta_{p}^{2}=0.019$; in contrast, there was a pronounced effect of LBR, *F*_2.82, 952.74_ = 383.11, *p *< 0.001, $\eta_{p}^{2}=0.531$ (here and elsewhere, a Huynh–Feldt correction was applied because of violations of sphericity [58]). The effects of LBR did not depend on ABR, *F*_15.50, 5237.87_ = 1.13, *p* = 0.323, $\eta_{p}^{2}=0.003$, or IR, *F*_5.64, 952.74_ = 0.38, *p* = 0.880, $\eta_{p}^{2}=0.002$; similarly, the effects of ABR were not modulated by IR, *F*_81_ _352_ = 1.33, *p* = 0.223, $\eta_{p}^{2}=0.008$, and there was no three-way interaction *F*_30.99, 5237.87_ = 1.19, *p* = 0.216, $\eta_{p}^{2}=0.007$.

Following up the main effects of IR and LBR, *post hoc* pairwise comparisons indicated that although the baseline IR was more attractive than the −3 condition, this difference did not survive Bonferroni correction (adjusted *p* = 0.051); the +3 condition did not differ from either the baseline condition (adjusted *p* = 0.132) or the −3 condition (adjusted *p* = 1.000). For LBR, all conditions were different from one another (adjusted *p* < 0.001) apart from the −2 and +3 conditions (adjusted *p* = 1.000).

The relationship between limb ratio and attractiveness has previously been described well by a quadratic curve (e.g. [25]). We must be cautious about applying this approach here because we only tested a small number of ratios, but it is nonetheless instructive to fit quadratic regression curves of the form $J=B_{0}+B_{1}\;\text{Ratio}+B_{2}\;\text{Rati}{\text{o}}^{2}$ to the mean judgements for each condition. The blue lines in figure 2 show the resulting curves. For IR, there are only 3 data points so the curve fits perfectly. For the ABR, the curve is essentially flat, with linear and quadratic terms that are very close to zero, consistent with the results of the ANOVA: *B*_0_ = 3.96, CI_95%_ = [3.91, 4.02], *p* < 0.001; *B*_1_ = −0.01, CI_95%_ = [−0.02, 0.01], *p* = 0.209; *B*_2_= −0.01, CI_95%_ = [−0.02, 0.00], *p* = 0.082; overall model *F*_2,2_ = 7.04, *p* = 0.124, $R_{\mathrm{adj}}^{2}=0.\text{75}$. For the LBR, there is a pronounced curvature to the judgement function, with a peak that is somewhat above the baseline *B*_0_ = 4.90, CI_95%_ = [4.56,5.23], *p* < 0.001; *B*_1_ = 0.19, CI_95%_ = [0.11, 0.27], *p* = 0.010; *B*_2_ = −0.19, CI_95%_ = [−0.24, −0.13], *p* = 0.004; overall model *F*_2,2_ = 162.8, *p* = 0.006, $R_{\mathrm{adj}}^{2}=0.\text{99}$. Differentiating and rearranging, the maximum attractiveness occurs when Ratio = 0.50, that is, when the LBR is half a standard deviation above the population mean, which is virtually identical to the findings of Versluys & Skylark [25].

In short, Study 1 replicated previous findings that attractiveness is influenced by LBR, with an optimum that is slightly above the population average. However, we found no effect of ABR and only a small effect of IR, and no indication that any of the three ratios modulated one another's effects. Study 2 was conducted in order to expand on these findings.

## 3. Study 2

The number of LBR, ABR and IR values used in Study 1 was constrained by the factorial design. Having found no indication of interactions between the effects of the three ratios, Study 2 was designed to provide a more thorough investigation of the main effect of each ratio. Correspondingly, participants were randomly assigned to judge male figures that differed either in LBR, ABR, or IR, and saw figures that were −3, −2, −1, 0, +1, +2, or + 3 s.d. from the mean.

### 3.1. Methods

#### 3.1.1. Participants

The sample comprised 253 heterosexual women aged 20–71 (*M* = 34.48, s.d. = 9.90); ethnicities: White (83.0%); Black (7.5%); Asian (4.7%); all others (4.7%).

#### 3.1.2. Stimuli

The stimuli were constructed as before. For each ratio (LBR, ABR and IR), figures were constructed that were −3, −2, −1, 0, +1, +2 or +3 s.d. from the baseline. As for Study 1, the manipulation of IR was conducted for both arms and legs simultaneously.

#### 3.1.3. Design and procedure

The study had a 7 (ratio) × 3 (limb variable) design in which participants were assigned randomly to either the LBR (*N* = 83), ABR (*N* = 83), or IR (*N* = 87) groups, viewing a total of 7 stimuli. These samples gave 97% power to detect a small effect ($\eta_{p}^{2}=0.02$). The procedure was the same as for Study 1.

### 3.2. Results and discussion

The mean judgements are plotted in the right column of figure 2. There was no effect of ABR on attractiveness judgements, *F*_5.60,459.49_ = 0.34, *p* = 0.904, $\eta_{p}^{2}=0.004$. However, as for Study 1 there was an effect of IR, *F*_6,516_ = 2.86, *p* = 0.009, $\eta_{p}^{2}=0.032$, and of LBR, *F*_5.01, 410.51_ = 35.27, *p* <  0.001, $\eta_{p}^{2}=0.301$. A 2 (limb type: arms versus legs) × 7 (ratio) mixed ANOVA revealed a substantial interaction, *F*_5.41, 886.70_ = 24.55, *p *< 0.001, $\eta_{p}^{2}=0.130$, confirming that the effect of changes in leg length was different from the effect of changes in arm length. Likewise, the effect of changing the IR was different from the effects of changes in LBR, *F*_5.60, 941.45_ = 17.92, *p *< 0.001, $\eta_{p}^{2}=0.096$, and ABR, *F*_6,1008_ = 2.41, *p* = 0.026, $\eta_{p}^{2}=0.014$. Taken together, these results demonstrate a pronounced effect of changes in LBR, a weaker effect of changes in IR and no effect of changes in ABR.

The blue lines in figure 2 show the best-fitting quadratic curves, computed as for Study 1. For the IR judgements, the quadratic curve provides a poor description of the data, $R_{\mathrm{adj}}^{2}=-0.0\text{13}$, *F*_2,4_ = 0.96, *p* = 0.456; *B*_0_ = 4.80, CI_95%_ = [4.56, 5.03], *p* < 0.001; *B*_1_ = 0.02, CI_95%_ = [−0.06, 0.09], *p* = 0.613; *B*_2_ = −0.02, CI_95%_ = [−0.06, 0.02], *p* = 0.272. Inspection of figure 2 suggests that the effect of IR may be better described as a sharp peak at the baseline ratio, with ratios that are more than one s.d. either side being judged less attractive and similar to one another, although the only *post hoc* comparison that survived Bonferroni correction was the difference between the baseline and −2 s.d. conditions (adjusted *p* = 0.021; all other adjusted *p* > 0.10).

As is clear from inspection and from the ANOVA, the curve is flat for the ABR judgements, $R_{\mathrm{adj}}^{2}=0.\text{15}$, *F*_2,4_ = 1.53, *p* = 0.321: *B*_0_ = 4.77, CI = [4.70,4.84], *p* < 0.001; *B*_1_ = −0.00, CI = [−0.03, 0.02], *p* = 0.889; *B*_2_ = 0.01, CI = [−0.01, 0.02], *p* = 0.156.

For LBR, the mean judgements are well described by a quadratic curve, $R_{\mathrm{adj}}^{2}=0.\text{88}$, *F*_2,4_ = 22.64, *p* = 0.007, *B*_0_ = 5.10, CI = [4.72, 5.48], *p* < 0.001; *B*_1_ = 0.11, CI = [−0.01, 0.23], *p* = 0.071; *B*_2_ = −0.16, CI = [−0.23,−0.09], *p* = 0.003. From the coefficients, the estimated optimum LBR is 0.34 s.d. above the baseline—similar to the value of 0.50 found in Study 1. Using the bootstrapping approach described by Versluys & Skylark [25], the 95% percentile-based confidence interval for the optimum LBR was [0.165, 0.520], confirming that the optimally attractive leg length is above the baseline. (The bias-corrected accelerated confidence interval is virtually identical.)

## 4. Study 3

Studies 1 and 2 both found a strong effect of LBR and no effect of ABR. They also found a weak effect of IR, when IR was manipulated for both limbs simultaneously (such that, for example, a high IR meant relatively long distal portions of both arms and legs). In Study 3, we probed the effect of IR in more detail by separately manipulating the IR of arms and legs.

### 4.1. Methods

#### 4.1.1. Participants

The sample comprised 193 heterosexual women aged 20–74 (*M* = 36.49, s.d. = 11.68); ethnicities: White (73.1%); Black (11.4%); Asian (5.2%); all others (10.4%).

#### 4.1.2. Stimuli

The stimuli were constructed as for Studies 1 and 2. Separately for both arms and legs, we constructed stimuli with IRs that were −3, −2, −1, 0, +1, +2 and +3 s.d. from the baseline.

#### 4.1.3. Design and procedure

The study had a 7 (IR) × 2 (limb) design; each participant was assigned randomly to one limb condition (arms, *N* = 96; legs, *N* = 97), giving 99% power to detect an effect of $\eta_{p}^{2}=0.03$ (the size found in Study 2). Each participant judged the 7 IR values, following the same procedure as for Studies 1 and 2.

### 4.2. Results and discussion

**Figure 3.**
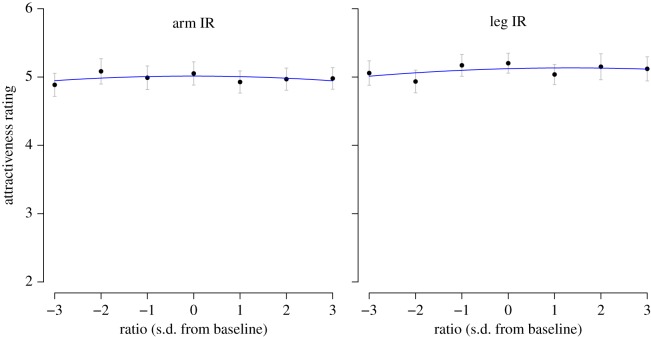
Results of Study 3. The left panel shows the results when the IR was varied for arms; the right panel shows the results when it was varied for legs. The plotted points show the mean attractiveness rating for each condition; error bars show 95% confidence intervals, computed for a within-subject design [57]. The blue lines show the best-fitting quadratic curves.

The mean judgements are plotted in figure 3; there is very little indication of a meaningful effect of IR on attractiveness judgements, both for the condition where IR was varied for arms and where it was varied for legs. This impression was supported by a 2 × 7 mixed ANOVA. As one would expect, there was no main effect of limb (that is, no difference in the overall attractiveness ratings for figures in which the IR was varied for legs rather than for arms), *F*_1, 191_ = 0.96, *p* = 0.330, $\eta_{p}^{2}=0.005$. More importantly, there was no main effect of IR, *F*_6, 1146_ = 0.874, *p* = 0.513, $\eta_{p}^{2}=0.005$, and no interaction between IR and limb, *F*_6, 1146_ = 0.98, *p *= 0.437, $\eta_{p}^{2}=0.005$.

The blue lines in figure 3 show the best-fitting quadrative curves through the mean judgements for each condition. Consistent with the ANOVA, the curve was flat for arms, $R_{\mathrm{adj}}^{2}=-0.\text{24}$, *F*_2,4_ = 0.42, *p* = 0.683, *B*_0_ = 5.01, CI = [4.89,5.14], *p* < 0.001; *B*_1_ = −0.00, CI = [−0.04, 0.04], *p* = 0.981; *B*_2_ = −0.01, CI = [−0.03, 0.02], *p* = 0.412. The same was true for legs, $R_{\mathrm{adj}}^{2}=-0.\text{15}$, *F*_2,4_ = 0.60, *p* = 0.591, *B*_0_ = 5.12, CI = [4.96,5.28], *p* < 0.001; *B*_1_ = 0.02, CI = [−0.03, 0.07], *p* = 0.410; *B*_2_ = −0.01, CI = [−0.04, 0.02], *p* = 0.582.

In short, when IR is manipulated for each limb separately, there is no indication that it affects attractiveness.

## 5. General discussion

Several important findings emerge from these studies. First, there is a clear preference for LBRs that are approximately 0.3–0.5 s.d. above the mean, with proportions becoming less appealing as they deviate from this point. This replicates the findings of Versluys & Skylark [25] and is similar to the preference for male LBRs that were 5% greater than baseline reported by Sorokowski & Pawlowski [20], and is consistent with the idea that leg-length preferences reflect a trade-off between the advantages of averageness, namely genetic diversity and associated immunocompetence, and those deriving from above-average size. In particular, relatively long legs are indicative of greater overall size, high socioeconomic status and biomechanical efficiency. In contrast, the only other study to have used 3D-rendered figures found that the average was optimally attractive [27]. The difference between these two sets of results may reflect small differences in stimulus format, which have been shown to moderate the effects of morphology [25,59]. They could also be caused by variation in participant characteristics in terms of sex, age, ethnicity or, more importantly, cultural differences: the present study was conducted with a sample of heterosexual females from the United States, whereas Kiire [27] used a combination of male and female undergraduates from Japan, a country with standards of attractiveness and anthropometry that are considerably different from those of most Western countries.

Second, we found no evidence that arm length influences attractiveness judgements: despite being highly powered, neither Study 1 nor Study 2 found evidence of a meaningful effect of ABR, and Study 2 confirmed that the effect of ABR was significantly less than that of LBR. It is important to note that our stimuli were chosen to be a given number of s.d. below/above the population mean; correspondingly the manipulation of LBR and ABR was functionally equivalent (i.e. ceteris paribus, there is equal probability of a female encountering a prospective mate with each of the tested morphologies). Thus, our results suggest that arm length is a relatively unimportant contributor to male attractiveness for the US population.

This finding presents a theoretical challenge, because many of the arguments that have been developed to account for effects of leg length should also apply to arms. In particular, the fitness benefits of averageness (as a signal of immunocompetence) and of above-average size (as a signal of reserve capacity) are both potentially applicable to ABR. A straightforward explanation is that the changes in ABR were less perceptually salient or detectable than the changes in LBR: the population variance is greater for legs than for arms, so the LBR stimuli differed more than the ABR stimuli (e.g. the maximal difference in ABR values in Study 2 was 0.063, whereas for LBRs it was 0.088; table 1). Another, not necessarily exclusive, possibility is that ABR may be a less meaningful signal of fitness. The relatively low variability in ABR may suggest that this aspect of morphology is subject to tighter constraints than leg length, limiting its role in sexual selection. With this in mind, it is notable that although arm length has been associated with a range of fitness indicators such as socioeconomic status and cognitive decline, the evidence is weaker than for LBR. A further possibility is that the relative contributions to attractiveness of LBR and ABR reflect transitory/cultural influences, since many factors other than biological fitness affect morphological preferences. Whatever its cause, the indifference of our participants to changes in ABR suggest that this aspect of morphology is not currently an important determinant of male reproductive success for this population.

The third contribution of our work is that it provides the first evidence that attractiveness is influenced by the ratio of the lower-to-upper limb segments: we found a slight preference for IRs that approximate the population average, with relatively low and high ratios being less appealing. This effect emerged in Studies 1 and 2, and is consistent with prior theoretical and empirical work suggesting that averageness signals fitness (e.g. [60]). However, the effect was relatively weak and, perhaps for this reason, was not well described by a quadratic curve; rather, there was a sharp peak at the mean and relative insensitivity to departures either side. Indeed, when IR was varied for just one limb type (Study 3), there was no longer any effect at all, despite the study having 99% power to detect an effect like that in Study 2, which probably reflects the fact that participants found it more difficult to detect IR variation in only a single limb than in both limbs together. This is especially likely given the relatively muscular physiques of the stimuli, which might have diverted attention away from changes in IR; more generally, it is clear from figure 1 that the changes in IR are, like the changes in ABR, hard to detect—despite spanning a range from the lowest 0.1% of the population to the highest 0.1%. Thus, the slight preference for average IR broadly accords with the negative fitness correlates of short distal limbs [30,44,45,49], but, like for arm length, our data suggest that the ratio of the lower-to-upper-limb segments is not taken as a very important signal of fitness and has limited effect on mate choice.

We have focused on the preferences of heterosexual women for male figures; clearly, it will be important to generalize our approach to test the preferences of male judges, and to examine preferences for female figures (e.g. [20,27]). Future work should also test whether our findings generalize across changes in cultural, social and design conditions, which have been shown to modulate preferences for other aspects of morphology (e.g. [25,61]). The current research, along with other recent work [25,27], employed several methodological innovations that will be useful in pursuing these future directions. In particular, the use of advanced CGI software and reliable anthropometric data, the selection of stimuli based on population standard deviations, and a curve-fitting approach to data analysis will all facilitate the development of precise, valid, and comparable measures of the consequences of limb variation in different populations and under different circumstances. Nonetheless, our studies have several limitations. We used a single ‘base’ body from the modelling software, and this physique is in some respects atypical (e.g. the figure is very lean and probably more attractively proportioned than the average man). Likewise, although we used an anthropological database to select limb proportions, the soldiers measured for this database are likely to differ from the overall population in some respects because of the fitness requirements for military service. Clearly, there is scope for future studies to use improved measurement data and a wider variety of base figures, to test the generality of the effects of limb proportions.

Taken together, our results add to the growing evidence that limb variation influences aspects of human well-being ranging from mate choice to employment prospects to medical outcomes, and also offer insights into the role of limb proportions in evolutionary history. In particular, the present data suggest that sexual selection might have acted (or be acting) differently on the arms and legs, with only the latter playing an important role in determining sexual selection. This is consistent with recent work showing that the genetic regulation of the front and hind limb diverged around the time of the Last Common Ancestor, which would have allowed for the possibility of differential selection pressures [62]. The present studies suggest that the behavioural and psychological implications of the genetic decoupling of the front and hind limb warrant further investigation.

## Supplementary Material

Explanatory notes for data for Versluys, Foley, and Skylark

## Supplementary Material

Data for Study 1 of Versluys, Foley, and Skylark

## Supplementary Material

Data for Study 2 of Versluys, Foley, and Skylark

## Supplementary Material

Data for Study 3 of Versluys, Foley, and Skylark

## Data accessibility

The data reported in this paper are included as electronic supplementary material.
